# The Address before the International Hahnemannian Association, June, 1884

**Published:** 1885-01

**Authors:** George F. Foote

**Affiliations:** President


### THE ADDRESS BEFORE THE INTERNATIONAL HAHNEMANNIAN ASSOCIATION, JUNE, 1884.

GEORGE F. FOOTE, M. D., PRESIDENT.

GENTLEMEN OF THE INTERNATIONAL HAHNEMANNIAN ASSOCIATION: We are now entering upon the fifth year of our existence as a body politic, and our success seems assured; allow me, therefore, to congratulate you, and with you rejoice at the success that is crowning our efforts to sustain pure Homœopathy. The need for this organization had become a necessity with those who regarded association as a means of mutual improvement by an interchange of thought, the relation of experiences, and the promulgation of the truths, as taught us by that great and good man, Samuel Hahnemann.

The existence of this necessity was to many of the older members of the American Institute, who believed in the fundamental laws of Homœopathy, a painful fact. When the Institute departed from its first love and began to worship with those who acknowledge no law in medicine; when a new generation of members, tinctured with the follies of allopathy, sought to ignore the *law* as given to us through the instrumentality of Hahnemann, there was a rising to defend this law and to do honor to the man through whom it was made manifest.

We are here to-day, gentlemen, to sustain the cause of our early faith. We are homœopathists, and, like all others who follow in the footsteps of our great teacher, we can assume the name without pretense or simulation. We are the disciples of Hahnemann, not ashamed of our calling, but proud of the profession through which we have been led into a calling that adds to the blessings of our fellow-beings.

In the name of the International Hahnemannian Association, I give you all a cordial greeting and a hearty welcome, and I commend each and all to the work in hand. Let each one feel a responsibility resting upon his own shoulders, and let each do his duty. Be vigilant and brave. Success will crown our efforts, as it ever does where truth prevails, though often at the expense of time.

The great question before you is, How can we, as an association, best promote the object of this combination? viz.: the promotion of our individual attainments as physicians under the great law of cure, *Similia, Similibus, Curantur,* together with the best means of promulgating these truths as to effect the greatest good to the greatest number.

There are many good men yet to be added to our fold; many good physicians who are anxious for the true light, but who have lacked the opportunity and experimental tests that we have enjoyed. To reach all such, with what we have to give, will be a good work and should become a part of the duties we impose upon ourselves.

The divisibility of matter and its development of potency as a source from which we obtain curative agencies, becomes a matter of interest, and though an old story to many of us, it is a subject that we may further investigate with profit.

Let we explain by a simple illustration. We will suppose I have here a cube of lead with its six surfaces, each with an area of just one square inch, in all six square inches. By cutting through each of these surfaces I shall have divided it into eight cubes, each of which will contain six superficial surfaces of one-half inch square, which, multiplied by the number of pieces, gives twelve square inches of surface.

Dividing these again as before, and we have sixty-four cubes, each surface of which measures three-eights of a square inch, with a combined surface of twenty-four square inches.

Another division, and we have five hundred and twelve pieces, with a superficial area of forty-eight square inches. The next division makes ninety-six square inches, and the fifth division makes one hundred and ninety-two square inches; and if continued to the tenth division, it gives to us nine hundred and two million one hundred and seventy-seven thousand two hundred and eighty minute cubes, with fifteen thousand three hundred and sixty square inches, or sixteen and six-tenths square feet of surface, which is two thousand five hundred and sixty times as many inches of surface as we had in the original cube.

Now, if we accept the theory that the toxic power of the lead, aside from its mechanical effect, depends upon the extent of the surface exposed, we have increased this toxic force by division two thousand five hundred and sixty times.

With this simple illustration we have a perception of what the division would have attained to had we made it with the centesimal instead of the octant scale, and we also have an apt illustration of the increased value of the toxic force by divisibility.

If the original cube of lead could have been taken into the stomach before its first division, there would have been but slight symptoms of lead poison, for the reason that only six square inches of its surface is exposed to contact with the surfaces of the alimentary canal. But with the tenth division by the octant scale, we have fifteen thousand three hundred and sixty square inches to be brought in contact with the surfaces of this canal, which, as before stated, is two thousand five hundred and sixty times greater than the surface exposed by the original cube of one inch, and, of course, must produce a corresponding increase of toxic effect.

But though we have from this single cube made over nine hundred millions of smaller cubes, all of them are too gross to be taken up by any of the vessels leading out of the alimentary canal. So we will now put it into a mortar and with the pestle crush each of these minute cubes into other millions of particles, or into an impalpable powder, with a corresponding increase of surface, and, of course, with a corresponding increase of toxic effect. And in this state of divisibility it can now be taken up and distributed to other parts of the system, dealing out a destructive force which in the original cube would be but slight, but now has accumulated to an agency sufficient to sicken and destroy the health, and perhaps the lives, of several people.

The questions now presenting themselves are: How is this effect produced by this agency? and, What becomes of this original lead when taken into the system? and also, What is its present relation to the system upon which it is operating with such destructive force?

The effects producible by the first divisions aside from what was physical would be scarcely perceptible. But with the tenth and beyond it is all powerful. A destructive poison and yet the original cube of lead, though not in taction as to its particles, is still in existence. It has not changed into any other substance, or rather, it has in no way changed its elementary character. It is still a metal. It may combine with other substances, but it is never annihilated. And after all its effects wrought upon the animal body it is still lead, and after its great work, could all its particles be aggregated, they would have a definite existence, as the metal lead corresponding in weight exactly to that of the original cube.

Here, then, is an effect without the loss of or change of substance, toxical, though indestructive to the material used. What, then, is the force developed? It is not mechanical; it is not physical; nor is it chemical. Therefore it must be something superinduced upon vitality by the mere presence of the lead. There is a force treated of in chemistry as applied to certain bodies that has a corresponding relation. This has been called catalysis. "A body having a force catalytique resolves other bodies into new compounds by mere contact or presence without itself experiencing any modification." But so far it has been a material presence of a tangible substance that has elicited force, and this is measured with a quantitative expression regarding the amount of material used. But experience teaches us that there is a higher power beyond this simple toxic effect obtainable through greater comminution. But we can no longer, after the few first divisions, carry it any higher without an intermediate, as the particles beyond a limited attenuation coalesce, and by the law of cohesion reunite so as to prevent further division. It is that attraction which takes place at insensible distances between homogeneous particles of bodies to form masses. So, as we advance in this division, we must insert some inert substance, as sugar of milk, by which we obtain a more diffusive and greater subdivision with a corresponding increase of potency. From this a much greater corresponding force is obtained in proportion to the amount of the original quantity of the lead used, as the intermediate substance also becomes imbued with this same power, which in itself becomes enlarged as we continue the subdivision. Hahnemann taught us that after the third centesimal trituration with the sugar of milk as a medium the metallic or mineral drug became soluble in alcohol or water, and he advised that further divisions should be made with these media.

Some of our eminent microscopists of to-day, failing to find particles of the metal visible with their highest objective glasses in the higher divisions, have cast a shadow of doubt upon this theory of Hahnemann. But so long as experience teaches us that this dynamic principle is communicated to the sugar of milk or to the liquid used, it is of little consequence whether any of the original particles of drug or metal used remain or not. We have gotten the force or potency sought for, which is sufficient.

Then, again, on the other hand, all crystallizable bodies are capable of a subdivision way beyond any development of the highest powers of the microscope.

In watching the crystallization of a solution of camphor, as seen by the higher objectives of the microscope, the invisible particles first unite to form the visible ones, and these again to each other and to others of a little larger size, and they to still larger, so that these doubtful criticisms, based upon microscopic observations, have but little weight in counterbalancing the solid facts obtained by many years of successful practice. Those of us who have ventured beyond the crudities of a toxic effect, those of us who acknowledge a higher law as taught by Hahnemann, know full well that there is an inner deep and lasting curative force that accompanies the potentialized medicines—a force visible only in its effects, an imponderable something that as a curative agent finds no parallel with that obtained from and by the administration of the crude drug.

Our successes in the cure of the sick depend, as a *sine qua non*, upon the quality of our medicines, and the importance of having these absolutely pure is a matter pregnant with vital interest to every practitioner. In using the higher attenuated preparations, the question is often raised, Can the menstruum used in diluting affect in any way the purity of the drug when it contains within itself other foreign matter?

This subject was discussed with some manifest interest at the last meeting of the American Institute.

Our late Dr. Joslin has left us as a legacy an analysis of this question that would seem to solve the whole matter and relieve us of all anxiety or doubt in reference to it.

His mathematical mind, with the aid of the microscope, enabled him to fathom this question and place it within the comprehension of every one.

He tells us that all bodies in a state of solution are converted into solid masses by the invisible particles first uniting to form visible solids, and that when these were developed and brought in contact with the same substance in a still less divided state, they unite with the latter and become more crude and unattenuated, and that this union by cohesion always takes place when one portion of the substance is only a hundredth part more diluted than the other. Thus a substance which happens to adulterate the menstruum used for attenuating a medicine is continually combining with its cruder particles and practically nullifying itself, while the higher potentized medicine remains practically pure.

He further tells us that these conditions of this reunion is not a mere hypothesis, but are in accordance with observed facts in crystallization.

The aggregation was never by the attachment of the infinitesimal to large masses, but to those within the sphere of the centesimal sizes, as when one solid joins itself to a solid not more than one hundred times larger than itself.

It is a singular coincidence, and almost more than an accidental fact, that Hahnemann should have selected this same centesimal scale in the attenuation of medicines to develop their potential powers as curative agents. And the idea is at least suggestive of a valid reason for confining our attenuating scale while developing these powers to this same multiple.

By the division of solids throughout a liquid there are two forces brought into activity—first, the affinity existing between the liquid and the solid masses, by which the latter are divisible into invisible solids or molecules; second, the cohesive force that tends to unite molecular solids of a homogeneous nature into massive proportions. These are antagonizing powers, and are both in the constant effort to establish an equilibrium by which these infinitesimal solids are held at equal distances from each other, of equal size and in a state of rest.

These forces play an important part in the attenuation of medicines while we are developing their potential capacities. Upon these activities we are largely indebted for their purity.

By the process for attenuating with liquids, should the original drug in the first dilution, be it in the form of a tincture, infusion, or a decoction, have been prepared with a menstruum free from all impurities, and the same kind of menstruum be used in the succeeding dilutions, properly manipulated in clean vials, the presumption is that our medicine is pure, with its potency duly developed.

Should the original drug or the first dilution thereof contain any impurities, and the subsequent attenuations be made with a menstruum that is pure, the impurities of the drug therein will be attenuated with the drug, and our medicine will be impure and unreliable.

Should the drug be originally prepared by the same impure menstruum that is used in its subsequent dilutions, and should these impurities be not homogeneous to the drug, therefore without that attractive force tending to molecular union, the drug alone will be attenuated. The impurities being alike in the liquid holding the medicine and the menstruum used, the equilibrium of their molecules are not disturbed by the intermixture, therefore they are not attenuated.

Should the original pure drug be prepared with a menstruum that is pure, and the succeeding dilutions with a menstruum that is impure, then, in the first dilution, the original solids of the drug will be divided and diffused throughout the ninety-nine drops of the added menstruum, while the impurities of the menstruum will be but slightly divided by the addition of the one one-hundredth part more of the pure menstruum in the drop containing the drug.

In the next dilution, the molecules of the slightly attenuated impurities, induced by the pure solvent accompanying the medicated drop used, will, by the force of cohesion that unites homogeneous solids, join the cruder molecules of the menstruum and become unattenuated. In the further dilutions of this same drug, while it continues to be attenuated with its potency developed, there will be no attenuation of the impurities, on account of the equality of the sizes and proportions of their molecules held in solution by the medicated liquid and the menstruum used.

Again, the question of purity, when medicines are attenuated by water containing foreign matter, as all waters do, is simplified, when that used in diluting is all from the same source. If the original medicine is pure and but one kind of menstruum is used, the process of diluting will affect only the medicine, for the reason that the impurities in the menstruum are already divided to the full capacity of the water. Adding from the same fountain water to water does not change the relation of their impurities, while the medicine continues to be divided with each addition of water and it alone becomes attenuated, and therefore potentized to a discreet degree above the contaminating influences of the impurities in the menstruum used.

Should the three first attenuations of a drug be made with sugar of milk originally impure, or made so by detritus from the mortar used, and thereafter attenuated with a liquid that is pure, the impurities of the sugar of the milk, being homogeneous to each other, coalesce as in the case of liquids, and in the third attenuation they are still crude and insoluble in the menstruum used. The drug, being pure and attenuated to the third centesimal degree, is soluble, and the succeeding attenuations, *ceteris paribus*, will be pure.

Should the attenuations made with the sugar of milk be further attenuated with a liquid containing impurities, the soluble drug alone will be attenuated pure and its potence developed, for reasons heretofore explained.

A very reprehensible system is that practiced by some physicians and others preparing the medicines, viz.: the pouring of a diluting liquid, be it alcohol or water, from a bottle into a vial that has been medicated, or that contains medicine. For the reason, that as the dilutent is poured from the bottle, there is a tendency to a vacuum within, into which the air readily flows as it is forced from the vial by the inflowing liquid. This transmitted air is impregnated with the aura vitalis of the medicine, which is communicated to the liquid within the bottle. To use this same liquid as a dilutent to other potencies or for other medicines, is simply mixing our drugs, and in the end producing a conglomerated mass of different medicines, thereby nullifying the best endeavors to prescribe *secundum artem*.

In accordance with these facts, the following rules should be strictly adhered to while manipulating drugs to develop their potencies by attenuations.

*First*. The drug to be attenuated must be pure.

*Second*. But one kind of menstruum, and all from the same source, should be used for one drug throughout the entire process.

*Third*. The menstruum used, be it alcohol or water, should never be poured from a bottle into a vial or other bottle containing medicine, or that has contained it and not thereafter purified, but always into clean vials, pouring the menstruum in first and the medicine afterward.

*Fourth*. After being used, all vessels should be exposed to a moderate red heat before using again, which is easily done with the aid of an alcohol lamp.

Before the days of Hahnemann the profession had been dealing with the toxic power of the drug, an effect still sought for by the allopathic physician. But as homœopathists, we seek for a higher law of effect and its application to cure. We learn by a simple illustration that potency is developed by a comminution of the drug. And as we continue the division by the addition of an intermediate, experience has taught us that we add, by the greater attenuation, to this potency a force hitherto unknown, or, at least, unused, until discovered and applied to use by Hahnemann. By this dynamic operation, we come under the domain of this higher law. Step by step, we pass from the crude toxic to an exalted force, from the material to the imponderable.

Hahnemann, in obtaining his thirtieth potency, filled thirty clean vials with alcohol: placing his drop of the crude tincture in the first vial, he gave it two sharp, concussive shakes, and thus obtained his first attenuation. One drop of this, placed in the second vial, with two similar shakes, makes the second attenuation, thus repeating the same from vial to vial, up to the thirtieth. Before using any of these vials for other medicines, they were exposed to a temperature nearly that of a red heat, to destroy any drug impression left upon or within them.

The consequence was, that Hahnemann used only absolutely pure medicines, prepared by his own hands; and the successes attending his cures of the sick, with his thirtieth potencies, have no parallel in the general practice of the present day.

The fluxion potencies have been used in what was supposed to be a highly attenuated form. By this process a stream of running water with agitating force is allowed to pass into and out of a vial or capsule, into which a drop of the medicine had been placed. When a quantity of water equaling the amount used by Hahnemann to produce any desired attenuation has been thus used, the final vialful receives the corresponding number given to Hahnemann's centesimal dilutions, and this process is continued to the fancied numbers of millions, billions, trillions, etc. By this process, with due care, the medicines must be pure; and of their efficacy we can bear witness in many cases. But the uncertainty of the scale with which they are enumerated renders them unsatisfactory.

After a careful investigation of all that has been made public, both of the pros and cons, of this question, I am satisfied that there has been a grave mistake in the numercial estimates by the originators, and that the enumeration by millions, billions, trillions, quadrillions, and quintillions are but flights of the imagination, based upon this error.

As they add the first ninety-nine drops of the diluting liquid to the one drop of the tincture, it is plain that they have produced the first centesimal attenuation. Now, instead of using one drop of this to another ninety-nine drops of the liquid, they use the whole one hundred drops, adding only another one hundred to these, and this they call the second attenuation, and so on continuously. As a mathematical problem the absurdity has no parallel in figures; it is simply one to one, or equal to equals, instead of one hundred to one.

The plea that the water escaping from the vial as fast as it runs in modifies the results, only adds confusion, as it precludes any basis for a calculation and renders the subject more obscure.

While we have witnessed the grand curative effects of these medicines, we are conscious of the desire to know what number of the centesimal scale we are using, and the uncertainty is not pleasant. Of one thing we may be positive, and that is that we are not using medicines prepared by the centesimal scale, but by one that is far below it—indeed, quite low in the scale of potencies.

The introduction of new and varied systems of enumerating our attenuations adds confusion to an already well-established and satisfactory system designed and given to us by our great teacher, Hahnemann, and this without a possible benefit to the profession by a change.

*Cui bono?* Why all this labor and care in preparing our medicines? The ready answer is, Observation and experience teaches that this is the plan *par excellence.*

But there is another answer, that to some of us, at least, has a significance commensurate with this important question. As we call to mind the best appointed process of manipulation to develop the most efficient power for combating disease, our attention is arrested to the fact that there must be something within and above what we see as an ultimate, and this something is the possibility of a link in the great chain between cause and effect, between spirit and matter, operating through the higher plane of life.

In conclusion, gentlemen, while in no instance will this Association as a body be held responsible for the individual opinions of its members, for principles advocated or theories advanced at its stated meetings or at other times, unless through adoption by resolution and vote, yet upon all subjects of interest to the profession, so far as the Chair has a voice, perfect freedom and the utmost liberty will be allowed consistent with parliamentary usages and the object of the Association.

It is to be expected that we may differ in many points, and that the plane of thought where truth alone presides may be found at varying distances between us, to be reached from opposite points step by step through a free interchange of thought and polemic discussion.

Obedience to law in every particular leads to the most perfect freedom. We have but the one law of cure, with its three attributes, the similar remedy, the single remedy, and the potentialized remedy.

As a body of co-workers, we have entered into a solemn compact to advocate, revere, and conform to this law. We may view it from different standpoints, but it is the beacon light upon which we fix our gaze that leads to a common centre and a haven of deliverance and rest for the burden of our cares.

As an Association, we have been criticised by enemies without and by friends within. We may profit by all this, regardless of the incentives that prompt to complain. Let each charge be carefully weighed in the scale at the tribunal of conscience, and in the future strike the balance by amending all deficiencies.

Unkind shots from without are spent upon the bulwarks of our defenses and do us no harm, and if a battery is found to be unmasked from within, we scarcely need anticipate danger if in patience we possess our souls. To be criticised by the pen of an able writer from this same within, is not necessarily an indication of exterminating belligerency. We have only to peer into the distance, made dim by reason of the mists created from shot and shell that are thrown with an unsparing hand so thickly about us, and we may find pictured upon the mind's eye the simile of a venerable war-horse, with dilating eyes and expanding nostrils, pawing the very turf beneath his feet with impatient mien. The danger seems lessened as we are made conscious that an active life spent in defending the true faith from its numerous enemies, by an incessant warfare, has made the ear sensitive to the first approach of an enemy, and even to the tread of an imaginary foe. Let us remember that often our best friend is he who tries to tell us of our faults.

